# Supplementary figures and images for: Numerical methods for the detection of phase defect structures in excitable media
Source: PLoS One. 2022 Jul 12;17(7):e0271351. doi: 10.1371/journal.pone.0271351 (PMC9275727; doi:10.1371/journal.pone.0271351)

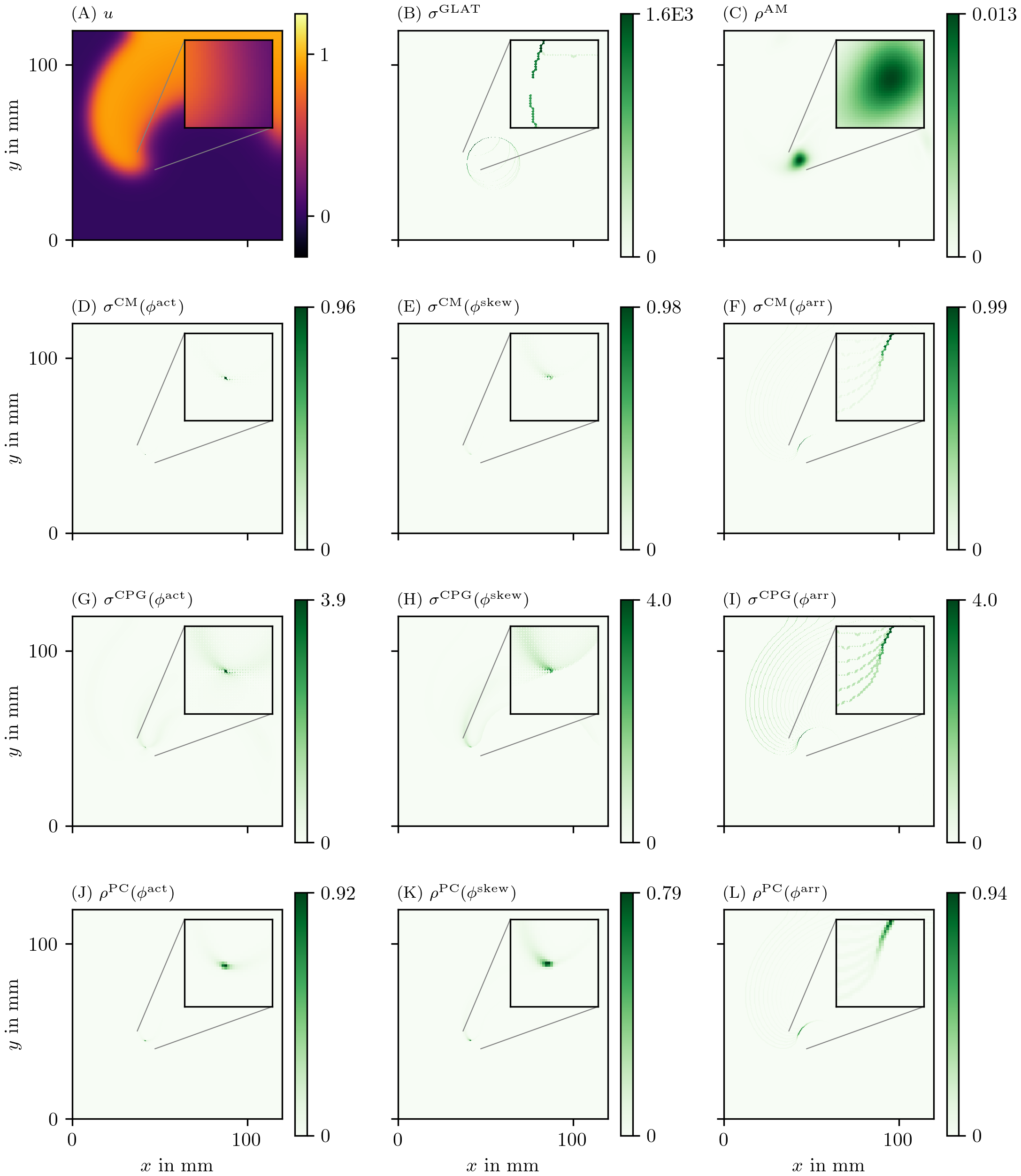

Supplement: S1 Fig — The data are presented in the same way as in Fig 6. (TIF) [file pone.0271351.s002.tif]

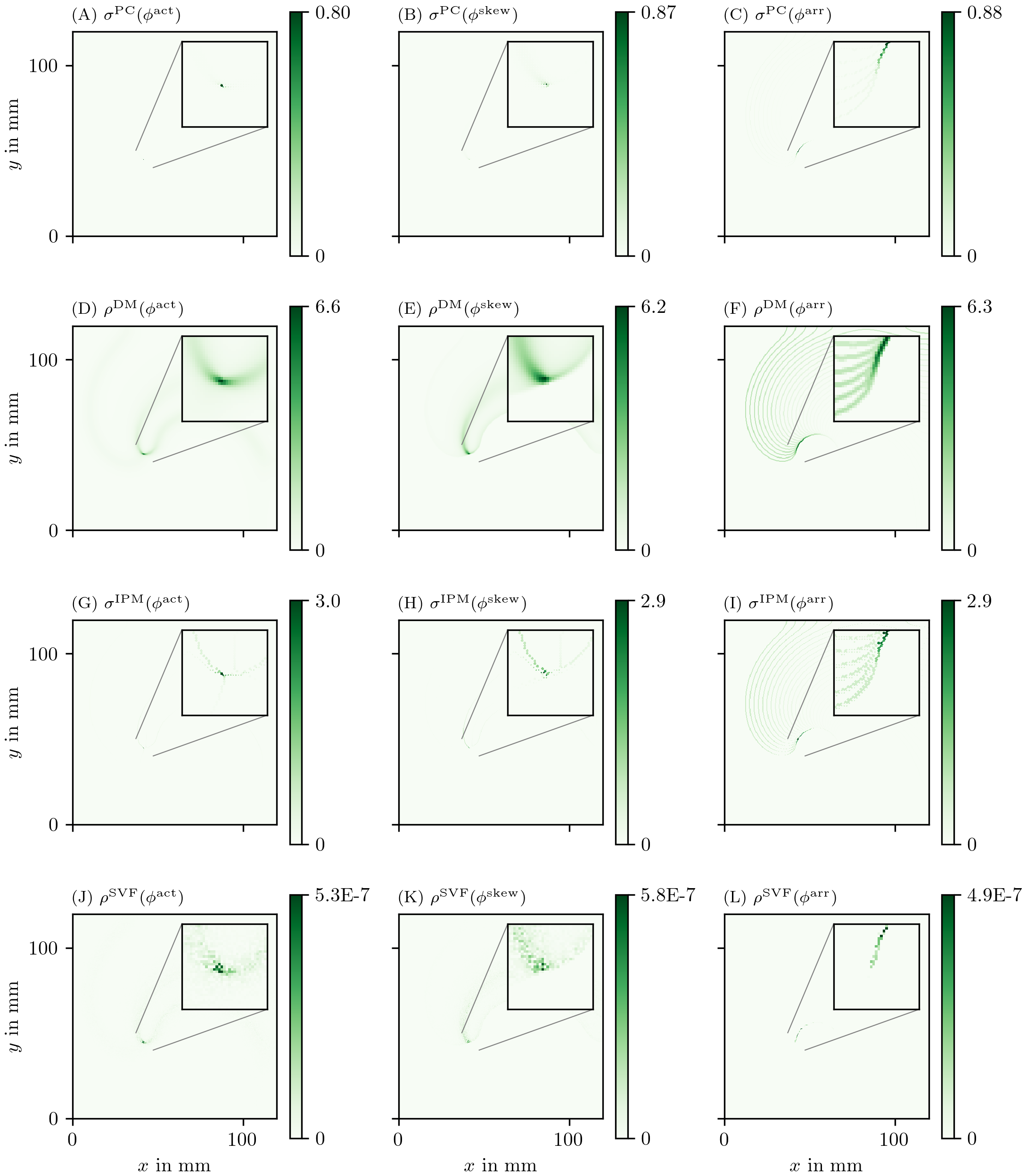

Supplement: S2 Fig — (TIF) [file pone.0271351.s003.tif]

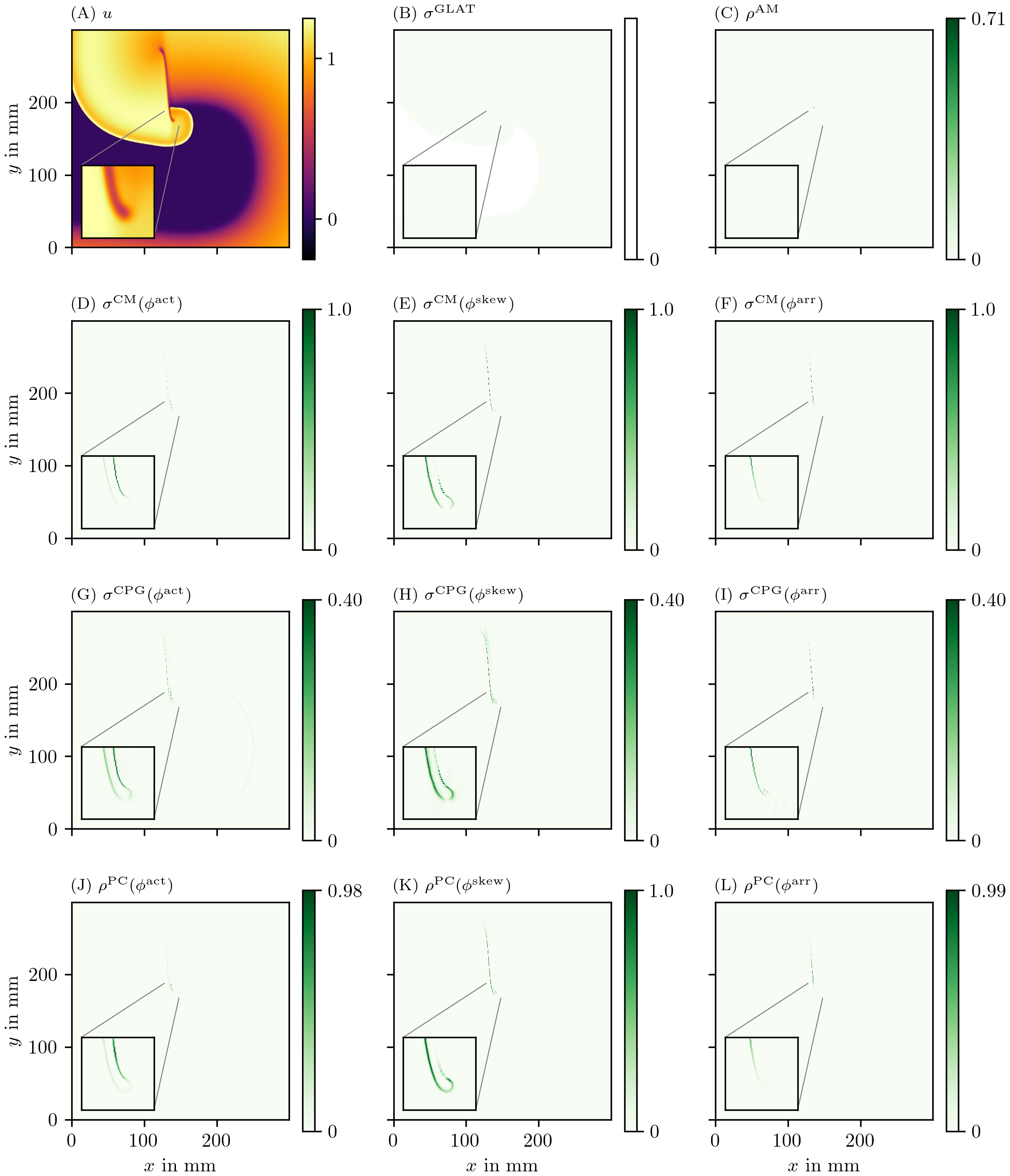

Supplement: S3 Fig — The data are presented in the same way as in Fig 6. (TIF) [file pone.0271351.s004.tif]

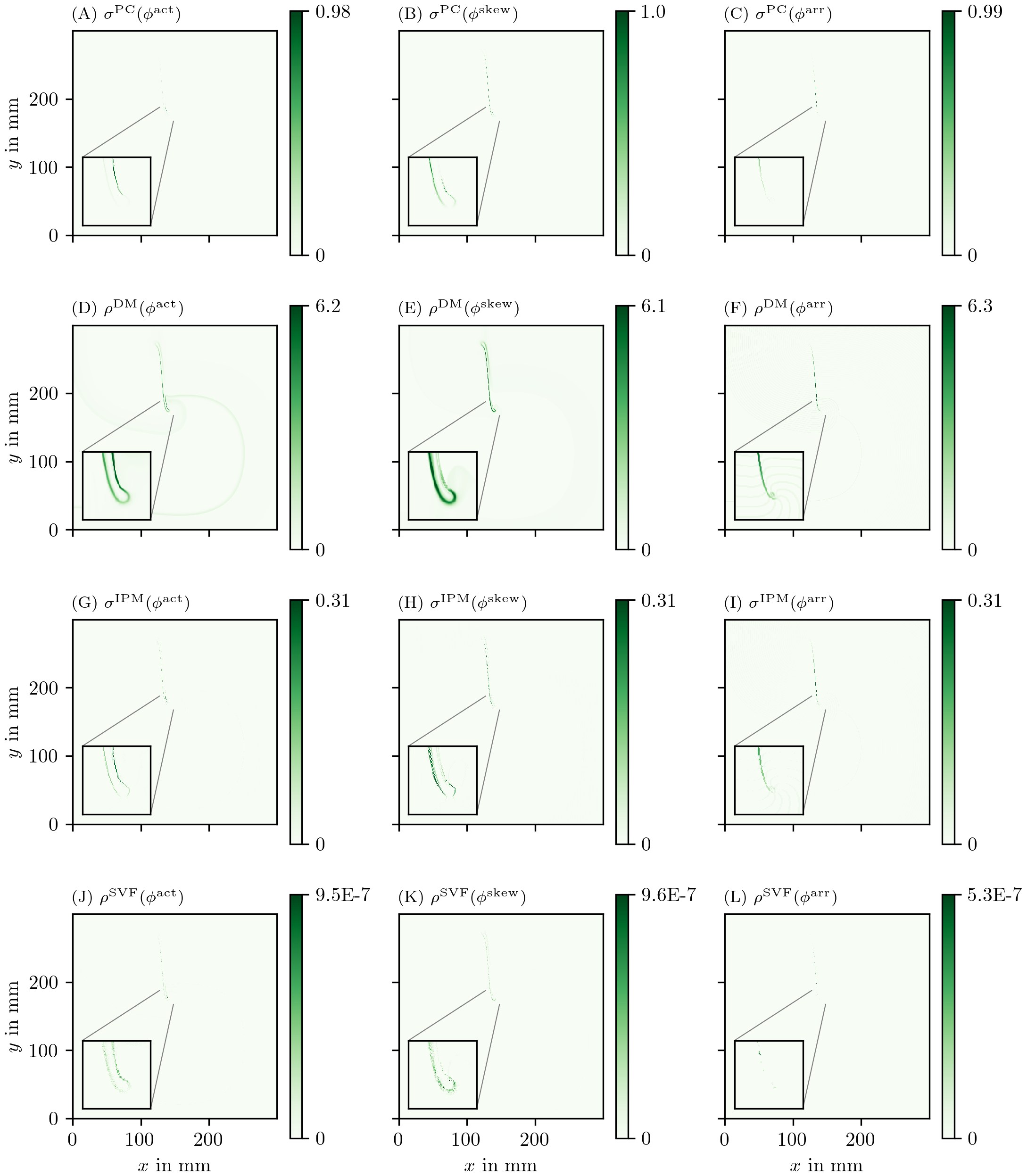

Supplement: S4 Fig — (TIF) [file pone.0271351.s005.tif]
