# Supplementary material for: Numerical methods for the detection of phase defect structures in excitable media
Source: PLoS One. 2022 Jul 12;17(7):e0271351. doi: 10.1371/journal.pone.0271351 (PMC9275727; doi:10.1371/journal.pone.0271351)
Supplement: Attachment — Submitted filename: responses.pdf [file pone.0271351.s006.pdf]

Responses to comments

Desmond Kabus et al.

2022-04-22

Reviewer #1:

> In this paper by Kabus et al, the idea of a phase defect line rather than phase singularities is explored, and methods for detection of these defects introduced and compared. The manuscript is well written, and the discussion and background on different methods is clear and very useful. All methods are freely provided and accessible which is also great.

Thank you for your feedback. It really helps.

> My only major comment is the results are currently lacking in my opinion, and this section could be extended. Extending the results however should be straightforward given the simulation and experimental data that already exists. With this, I think this manuscript will be very useful to many researchers.

We understand this point and have added further results in the new version of this paper. In future work, we will look at more experimental data, specifically optical voltage mapping data. In this paper however, we want to focus on in-silico experiments to show the feasibility of our algorithms.

> Results currently just show qualitive examples of the different methods, and the correlation statistics between them. However, I think simple experiments can be performed to compare the methods more thoroughly. For example, are there minimum criteria needed in terms of spatial and temporal resolution and are some methods 'better' at dealing with low resolution data. This has important consequences in which method to use for real life cardiac mapping data.

In our new version, we are investigating the effects of working at much lower resolution and noisy data on our algorithms.

> Could all these methods also be applied to extracellular recordings from mapping systems, as well as direct transmembrane data has done here? These studies are actually proposed in the discussion (line 406) but I think should be simple to perform now and really improve the paper.

As long as a phase can be defined, our methods can be applied to it. Specifically the elapsed time phase is well suited for such recordings, as one just needs to be able to measure local arrival time for it.

Minor comments:

> Overall, the figure quality does not look great to me and some parts are hard to see, but this may just be from embedding.

This is indeed an issue. We have supplied our figures in very high quality TIFF files at 600DPI to PLOS, however their converter to PDF seems to mess them up. We have fixed this issue in our preprint on bioRxiv and will discuss this issue with the publisher when we get closer to printing the final version.

> Figure 1: Can a scale be provided?

We have rescaled the data such that the resting state corresponds to a value of 0 and the plateau to 1. We use the same colouring in all plots and only provide a color bar in figure 2. In the updated version, we provide color bars for all sub plots, except for plots of phase as this colouring is cyclic and intuitive.

> Figure 2: Panel C hard to see, possibly rescale?

We have added insets to this figure like in the later figures of this paper. In the inset the phase defect can be seen better in that panel.

> Line 81: The definition of tau depends on the APD. Is this value therefore calculated at every point in the medium to account for heterogeneous APD in the tissue?

We determined a typical value for each data set / each used tissue model. We present this value in Table 1. For the optical voltage mapping, we use a typical value inbetween APD50 and APD80 as published by Harlaar et al. (2022). A note has been added to the manuscript to specifically mention this.

> Optical mapping methods are very sparse. What dye was used and how was it excited? Camera details are missing. 6ms is on the slow side for exposure time, is this a problem for phase mapping of optical tissue?

See the publication Harlaar et al. (2022) for the full protocols with which these data are obtained.

No, low time resolution is not an issue for this, see Gray, Pertsov, and Jalife (1998).

> Will the paper detailing the protocol for atrial differentiation be published soon/accessible to the reader? Either way, some brief methods should be included showing how the atrial cells were produced.

Yes, we are very happy that it is finally published in Nature Biomedical Engineering. (Harlaar et al. 2022)

> Line 191. It is asserted that there is no ground truth for testing PD as it is essentially where the chosen algorithm finds it. Could a simulation not be conducted where a ground truth is known however, for example an anatomical region that anchors a re-entrant wave?

Our methods find anatomical reentry around a line obstacle which will appear as a PDL when sampled at lower spatial sampling resolution. This experiment shows that the methods successfully recover such obstacles and therefore in a way validates them. Additionally, in the case of functional reentry, the methods described here show a phase defect at the rotor core. (Tomii et al. 2021; Arno et al. 2021)

> Line 300: ‘section 6,6,6 and 6’ not needed

We missed swapping the `\ref` command to the `\nameref` command here. It is fixed now. Sorry for the inconvenience.

Reviewer #2:

> In this paper, several different numerical methods to detect phase defects are presented. The methods were then tested using three computational models and an optical recording of a monolayer of human atrial myocytes.

First of all, thank you for your feedback. It is very much appreciated.

> Although the methods in the paper appear to be sound, the results are not particularly exciting. Most of the work is based on recent studies by the same group and this paper compares different methods of finding phase defect lines.

We appear to disagree here. The main goal was to find algorithms that can reliably find phase defect lines which we have achieved. In future works, we will use these methods to study phase defect lines in all sort of different experiments. We successfully demonstrate that our methods can be used for optical voltage mapping data for human atrial myocytes. Further studies will be carried out.

> Since there is not “ground truth”, it is not completely clear which one of these methods are the most promising ones.

That is very much a valid criticism. We have conducted an experiment where a ground truth is known and added it to the paper. We have placed an elongated obstacle at the centre of a rotor which is successfully detected by our methods.

> Furthermore, the numerical models used in the study are idealized since there is no noise or heterogeneities added to the model. I would have expected a more rigorous analysis that addressed how the methods perform when the data is noisier or when the spatial/temporal resolution is reduced.

In the latest version of the paper, we are now investigating the effects of working at much lower resolution and noisy data onto our algorithms. We show that the methods are robust to such settings.

> The one experimental application gives results that are hard to interpret. What to make of the results in Fig. 11 and 12?

We have added a more detailed conclusion in the discussion to address this issue.

All in all, I think this paper is better suited for a more specialized journal.

Thank you for your constructive feedback. We will move to PLOS One instead of PLOS Comp Biol.

I have a few additional comments:

The reference to the clinical paper (Ref. 9) that supposedly showed lines of block is not very convincing. First, the reader may think that the study was carried out in humans, especially since it was not grouped under the "experiments" reference (Ref. 8). However, it was not. Furthermore, the reentry in that study was achieved by creating an ischemic zone and not, as in the model, arising in homogeneous tissue. Finally, the reentry patterns obtained in the study did not clearly (at least not to me) show lines of block. I would advise to find a better reference or to clarify the relevance of this study.

Thank you for this comment, but we think this reference suits this context well. For instance, in Fig 10 of this clinical paper Janse et al. (1980), what we call "PDL" is denoted by the line following the "T" symbols.

Fig. 4 is not that clear when discussed on line 153. Please explain the vertical bands in a and c. Also, what does "darker shading corresponds to higher logarithmic probability density" mean? Maybe this is related to the discussion on line 290-294, where Fig.4 seems to be revisited. This is confusing to me.

We have modified this figure and added more explanation in the newest draft to make it hopefully easier to understand.

What does "work on data sets consisting out of phase values" mean? I don't understand the "out of phase" part.

This is indeed very confusingly written. We have fixed this in the current version.

Line 213: "and define rhoCM via Eq (15)". This doesn't make too much sense. Are the authors referring to an equation yet to come? If that is the case, do we need to replace the subscripts for it to make sense?

This has been fixed. The wrong equation was cited here.

line 300: section 6, 6, 6, and 6: please correct.

Done.

line 312: "slightly stresses the wave front" Not sure what that means. Can the authors explain?

This sentence has been rephrased to clarify.

> Figs. 9 and 11 do not seem to be described in the text.

We are now properly refering to both figures in the current draft.

> It would be nice to include movies of the results.

We are adding a selection of movies to the supplementary material of the next draft of this paper.

Reviewer #3:

> In this paper the authors compare a number of computational algorithms to detect phase defect lines, a generalisation of phase singularities commonly identified in the analysis of activation patterns in excitable media. Three definitions of phase are assessed using both simulated and optical mapping data. Some of the algorithms have some novelty, but most are algorithms from the literature, or adaptions thereof. The authors conclude in the end that phase definition is more important than the algorithm used and that the existing LAT-phase is the recommended choice.

> Strengths: The paper is generally well-written and well-structured and the analysis is systematic. The results support the conclusions. Open-source code is available for all the algorithms and figures.

> Weaknesses: No actual biological insight, limited significance and novelty, poor quality figures.

Thank you for your feedback. It is very much appreciated.

We acknowledge that this paper mostly contains tools and therefore mostly includes technical insights rather than biological ones. For this reason, we are now submitting to PLOS One instead of PLOS Comp Biol.

This manuscript is following up on a recent paradigm shift in the analysis of cardiac activation patterns that was initiated by Tomii et al. (2021) and Arno et al. (2021).

The present manuscript aims to split technical discussion from biological results, which we aim to publish in the near future.

Major Comments:

> There appears to be no biological insight from this paper, which seems to not be aligned with the journal's stated aim to further our understanding of living systems at all scales. From the conclusions, the outcome of this study does not seem to be sufficiently significant to warrant publication in PLOS computational biology.

Thank you for your constructive feedback. We will move to PLOS One instead of PLOS Comp Biol. We think that journal will be a better fit for this paper.

> New algorithmic contributions seem limited. The authors propose skewed phase, but this seems to be just a shift and renormalisation of activation phase. The authors appear to propose new phase detection algorithms, since they do not have corresponding citations to the literature, but this is not completely clear from the text. Some clarity on the specific contributions in the paper would be appreciated. In the end, the authors conclude that the definition of phase is more important than the algorithm.

We respectfully disagree here that our algorithmic contributions are limited. While in fact the skewed phase is just a transformed version of the activation phase, it does provide the useful insight that we can use this transformation to get rid of the sharp increase in phase at the wavefront. This is needed to ensure that the PDL is not located at the wave front but at the centre of the rotors. Other functions than the piecewise linear function can be used.

As for the phase detection algorithms, six of the nine algorithms presented here have to our knowledge not been published before.

We do however note that we can more clearly differentiate between our work and previous work. For this, we have added sources for the methods to Table 2. Every time we introduce an algorithm in the paper, we are clearly stating when it has been introduced elsewhere before.

> Resolution of all figures is generally poor and pixelated. In particular, there are significant compression artefacts around text/lines in figures, which makes the PD lines difficult to see and compare and some figure labels completely illegible, hampering interpretation of the data. Use of lossless tiff format would be better.

This is indeed an issue. We have supplied our figures in very high quality TIFF files at 600DPI to PLOS, however their converter to PDF seems to mess them up. We have fixed this issue in our preprint on bioRxiv and will discuss this issue with the publisher when we get closer to printing the final version.

> All comparisons are conducted on a regular grid (FD, optical mapping). In practice, the phase will often not be available on a regular grid (i.e. unstructured finite elements, or from electrogram measurements) and may only be available at a much coarser scale than the finite difference grid used in this study. The authors allude to this being future work, but this extension would significantly improve the impact of the paper and the usefulness of the results to the community. Such analysis might also help to differentiate the algorithms more.

All definitions have been formulated in a general way such that they can be used on non-regular grids. In our new version, we are investigating the effects of working at much lower resolution and noisy data on our algorithms. We hope these additions satisfy you to some extent.

> Why were significantly different grid sizes and time-steps used per model? This is presumably down to the stiffness of each model, but would this not affect the ability to resolve PDLs? Presumably a way to address this would be to interpolate all methods onto the coarsest grid? Would be reassuring to see evidence of convergence of the numerical method in an appendix. It would also be beneficial to list model/parameters explicitly in appendix, or give reference to exact table of parameters in original paper.

We performed studies on the effect of spatial resolution which shows that phase defects can also be detected on coarser grids.

Minor comments:

> L134: Why only a quarter of the domain stimulated if half the domain has repolarised? It is not clear from the description why that would produce a spiral wave and not just a second wavefront. Maybe the regions stimulated could be quoted explicitly?

We have rewritten this paragraph in a less ambiguous way.

> Fig 1 caption, AP, FK, BOCM not defined until later in the paper. OM not defined in caption. Colour bars missing. Sub-fig labels of right-column are incorrect (should be D, H, L, P).

This is fixed in the current version. We have added colour bars to all plots except phase plots which use a cyclic colour map.

> L149: Fig 1D should be Fig 1(M,N,O,P)?

This is fixed in the current version.

> Typo repeated words just before eq 8.

This is fixed in the current version.

> L202: For a 3D Cartesian grid, should Na not be 6, rather than 4 (which is the case in 2D)?

Indeed. This is fixed in the current version.

> Fig 3 Colour bars missing. Please include explicitly on each figure for ease of interpretation.

This is fixed in the current version. We have added colour bars to all plots except phase plots which use a cyclic colour map.

> L287: Would be helpful to indicate on the figure the PSs and extended phase defect being referred to to help the reader (improved figure quality may also help).

While the focus of this paper is on the PDLs, we agree that it would indeed be helpful to include PSs in some of the figure. In our latest version, we address this.

References:

Arno, L., J. Quan, N.T. Nguyen, M. Vanmarcke, E. G. Tolkacheva, and H. Dierckx. 2021. "A Phase Defect Framework for the Analysis of Cardiac Arrhythmia Patterns." *Front. Physiol.* 12. https://doi.org/%7B10.3389/fphys.2021.690453%7D.

Gray, R. A., A. M. Pertsov, and J. Jalife. 1998. “Spatial and Temporal Organization During Cardiac Fibrillation.” *Nature* 392: 75–78.

Harlaar, Niels, Sven O. Dekker, Juan Zhang, Rebecca R. Snabel, Marieke W. Veldkamp, Arie O. Verkerk, Carla Cofiño Fabres, et al. 2022. “Conditional Immortalization of Human Atrial Myocytes for the Generation of in Vitro Models of Atrial Fibrillation.” *Nature Biomedical Engineering*, January. https://doi.org/10.1038/s41551-021-00827-5.

Janse, M. J., F. J. L. van Capelle, H. Morsink, A. G. Kleber, F. J. G. Wilms-Schopman, R. Cardinal, C. Naumann D’Alnoncourt, and D. Durrer. 1980. “Flow of Injury Current and Patterns of Excitation During Early Ventricular Arrhythmias in Acute Regional Myocardial Ischemia in Isolated Porcine and Canine Hearts. Evidence for 2 Different Arrhythmogenic Mechanisms.” *Circulation Research* 47: 151–65.

Tomii, N., M. Yamazaki, T Ashihara, K. Nakazawa, N. Shibata, H. Honjo, and I. Sakuma. 2021. “Spatial Phase Discontinuity at the Center of Moving Cardiac Spiral Waves.” *Computers in Biology and Medicine* 130.
