# Supplementary material for: Numerical methods for the detection of phase defect structures in excitable media
Source: PLoS One. 2022 Jul 12;17(7):e0271351. doi: 10.1371/journal.pone.0271351 (PMC9275727; doi:10.1371/journal.pone.0271351)
Supplement: Peer review history

# Author comment: Author response to previous submission

**DOI:** 10.1371/journal.pone.0271351.r001

25 Apr 2022

Attachment Submitted filename: responses.pdf Click here for additional data file.

# Editor report: Decision Letter 0

**DOI:** 10.1371/journal.pone.0271351.r002

**Authors:** Ivan Kryven

29 Jun 2022

Numerical methods for the detection of phase defect structures in excitable media

PONE-D-22-12108

Dear Dr. Kabus,

We’re pleased to inform you that your manuscript has been judged scientifically suitable for publication and will be formally accepted for publication once it meets all outstanding technical requirements.

Within one week, you’ll receive an e-mail detailing the required amendments. When these have been addressed, you’ll receive a formal acceptance letter and your manuscript will be scheduled for publication.

An invoice for payment will follow shortly after the formal acceptance. To ensure an efficient process, please log into Editorial Manager at http://www.editorialmanager.com/pone/, click the 'Update My Information' link at the top of the page, and double check that your user information is up-to-date. If you have any billing related questions, please contact our Author Billing department directly at authorbilling@plos.org.

If your institution or institutions have a press office, please notify them about your upcoming paper to help maximize its impact. If they’ll be preparing press materials, please inform our press team as soon as possible -- no later than 48 hours after receiving the formal acceptance. Your manuscript will remain under strict press embargo until 2 pm Eastern Time on the date of publication. For more information, please contact onepress@plos.org.

Kind regards,

Ivan Kryven

Academic Editor

PLOS ONE

Journal Requirements:

When submitting your revision, we need you to address these additional requirements.

1. Please ensure that your manuscript meets PLOS ONE's style requirements, including those for file naming. The PLOS ONE style templates can be found at 

https://journals.plos.org/plosone/s/file?id=wjVg/PLOSOne_formatting_sample_main_body.pdf and 

https://journals.plos.org/plosone/s/file?id=ba62/PLOSOne_formatting_sample_title_authors_affiliations.pdf

2. Thank you for stating the following financial disclosure: 

"DK is supported by KU Leuven grant GPUL/20/012. LA was funded by a KU Leuven FLOF grant and a FWO-Flanders fellowship, grant 1177022N; LL was funded by KU Leuven and FWO-Flanders, grant G025820N. Research at Sechenov University was financed by the Ministry of Science and Higher Education of the Russian Federation within the framework of state support for the creation and development of World-Class Research Centers "Digital biodesign and personalized healthcare" 075-15-2020-926."

Please state what role the funders took in the study.  If the funders had no role, please state: ""The funders had no role in study design, data collection and analysis, decision to publish, or preparation of the manuscript."" 

If this statement is not correct you must amend it as needed. 

Please respond by return e-mail so that we can amend your financial disclosure and competing interests on your behalf.

3. Please include your full ethics statement in the ‘Methods’ section of your manuscript file. In your statement, please include the full name of the IRB or ethics committee who approved or waived your study, as well as whether or not you obtained informed written or verbal consent. If consent was waived for your study, please include this information in your statement as well. 

Additional Editor Comments (optional):

Reviewers' comments:

# Editor report: Acceptance letter

**DOI:** 10.1371/journal.pone.0271351.r003

**Authors:** Ivan Kryven

4 Jul 2022

PONE-D-22-12108 

Numerical methods for the detection of phase defect structures in excitable media 

Dear Dr. Kabus:

I'm pleased to inform you that your manuscript has been deemed suitable for publication in PLOS ONE. Congratulations! Your manuscript is now with our production department. 

If your institution or institutions have a press office, please let them know about your upcoming paper now to help maximize its impact. If they'll be preparing press materials, please inform our press team within the next 48 hours. Your manuscript will remain under strict press embargo until 2 pm Eastern Time on the date of publication. For more information please contact onepress@plos.org.

If we can help with anything else, please email us at plosone@plos.org. 

Thank you for submitting your work to PLOS ONE and supporting open access. 

Kind regards, 

PLOS ONE Editorial Office Staff

on behalf of

Dr. Ivan Kryven 

Academic Editor

PLOS ONE
